# University Students’ Self-Rated Health in Relation to Perceived Acoustic Environment during the COVID-19 Home Quarantine

**DOI:** 10.3390/ijerph18052538

**Published:** 2021-03-04

**Authors:** Angel M. Dzhambov, Peter Lercher, Drozdstoy Stoyanov, Nadezhda Petrova, Stoyan Novakov, Donka D. Dimitrova

**Affiliations:** 1 Department of Hygiene, Faculty of Public Health, Medical University of Plovdiv, 4002 Plovdiv, Bulgaria; 2 Institute for Highway Engineering and Transport Planning, Graz University of Technology, 8010 Graz, Austria; peter.lercher@tugraz.at; 3 Department of Psychiatry and Medical Psychology, Faculty of Medicine, Medical University of Plovdiv, 4002 Plovdiv, Bulgaria; drozdstoy.stoyanov@mu-plovdiv.bg; 4 Research Institute at Medical University—Plovdiv, 4002 Plovdiv, Bulgaria; 5 Department of Anatomy, Histology and Embryology, Faculty of Medicine, Medical University of Plovdiv, 4002 Plovdiv, Bulgaria; nadezhda.petrova@mu-plovdiv.bg (N.P.); stoyan.novakov@mu-plovdiv.bg (S.N.); 6 Department of Health Management and Healthcare Economics, Faculty of Public Health, Medical University of Plovdiv, 4002 Plovdiv, Bulgaria; donka.dimitrova@mu-plovdiv.bg

**Keywords:** environmental sensitivity, indoor environment, low frequency noise, nature sounds, self-rated health, soundscape, traffic noise

## Abstract

Background: Online education became mandatory for many students during the Coronavirus disease 2019 (COVID-19) pandemic and blurred the distinction between settings where processes of stress and restoration used to take place. The lockdown also likely changed perceptions of the indoor acoustic environment (i.e., soundscape) and raised its importance. In the present study, we seek to understand how indoor soundscape related to university students’ self-rated health in Bulgaria around the time that the country was under a state of emergency declaration caused by the COVID-19 pandemic. Methods: Between 17 May and 10 June 2020, we conducted a cross-sectional online survey among 323 students (median age 21 years; 31% male) from two universities in the city of Plovdiv, Bulgaria. Self-rated health (SRH) was measured with a single-item. Participants were asked how frequently they heard different types of sounds while at home and how pleasant they considered each of those sounds to be. Restorative quality of the home (the “being away” dimension of the Perceived Restorativeness Scale) was measured with a single-item. A priori confounders and effect modifiers included sociodemographics, house-related characteristics, general sensitivity to environmental influences, and mental health. Our analysis strategy involved sequential exploratory factor analysis (EFA), multivariate linear and ordinal regressions, effect modification tests, and structural equation modeling (SEM). Results: EFA supported grouping perceived sounds into three distinct factors—mechanical, human, and nature sounds. Regression analyses revealed that greater exposure to mechanical sounds was consistently associated with worse SRH, whereas no significant associations were found for human and nature sounds. In SEM, exposure to mechanical sounds related to lower restorative quality of the home, and then to poorer SRH, whereas nature sounds correlated with higher restorative quality, and in turn with better SRH. Conclusions: These findings suggest a role of positive indoor soundscape and restorative quality for promoting self-rated health in times of social distancing.

## 1. Introduction

In 2020, the world faced an unprecedented pandemic caused by the severe acute respiratory syndrome coronavirus 2. As a reaction, governments enacted anti-COVID measures to control the pandemic [1,2]. While effective in reducing Coronavirus disease 2019 (COVID-19) incidence and mortality, people’s lives were profoundly affected in various ways. Specifically, stay-in-shelter orders took a heavy toll on quality of life by adding the stress of social isolation [3,4]. Effective coping with everyday stressors requires periodic restoration, that is, renewal of adaptive capacities (cognitive, physiological, and social) diminished in ongoing efforts to meet adaptive demands [5,6]. However, opportunities to escape taxing demands were restricted because for many people the home environment, usually seen as a place for restoration, became a center of the same demands it used to provide respite from [7,8]. Teleworking and online education became mandatory for many and blurred the distinction between settings where processes of stress and restoration used to take place [9], thus likely compromising the restorative potential of the home environment for some population groups, such as university students [10].

Looking beyond this micro scale, however, one important side effect of the COVID-19 lockdown was the striking change in use of public spaces and transportation networks. The marked reduction in people’s mobility resulted in changes not only in air pollution [11,12], but also traffic noise exposure. Second only to particulate air pollution, traffic noise is an important environmental risk factor across Europe [13,14], implicated in increasing incidence of cardiometabolic diseases [15], sleep disturbance [16], depression and anxiety [17], adverse birth outcomes [18], and impairments of quality of life through noise annoyance [19] and interference with activities. In 2020, looming data suggest decreases in traffic noise levels in different cities during national lockdown periods [20]. For example, a study in Dublin found a significant reduction of about 5 dB in hourly average equivalent sound pressure levels and a several fold reduction in the time sound pressure levels exceed the regulatory threshold of 55 dB [21]. Another study observed an average of 3 dB reduction in personal sound exposure across several US states [22]. However, noise reduction differed across and within cities. A study in London observed a spatially heterogeneous noise reduction ranging from 1.2 to 10.7 dB [23]. This is not surprising, as noise levels in cities are determined by not only the amount of traffic, but also by urban morphology (e.g., land use, traffic network design, building layout and façade orientation, and green space ratio) [24,25,26]. Traffic flow composition was also altered. Heavy-duty vehicle traffic was affected much less compared with car traffic, owing to the need to maintain transportation of goods [27].

This observed change in traffic volume and type led to a shift in the balance between unwanted and wanted sounds, with likely impact on people’s health-related quality of life [28]. Restricting people’s activity spaces, the lockdown changed perceptions of the indoor acoustic environment (i.e., soundscape) and raised its importance as well [29,30]. On the one hand, exposure time to indoor sources of mechanical sounds increased during home confinement. A study reported that, during the lockdown in Turkey, participants’ annoyance by noise sources within their own dwelling increased, even though noise annoyance due to neighbors remained unchanged [31]. Prolonged exposure to low frequency sounds emitted by appliances, air conditioning, and ventilation systems can lead to annoyance and sleep-related problems [32,33]. On the other hand, the decrease in anthropogenic noise in cities unmasked and likely promoted the contribution to indoor soundscape of penetrating nature sounds from birds, water, and wind [34,35,36], which may confer psycho-physiological health benefits [37,38,39,40]. Indoor human sounds like familiar voices (e.g., of a family member or a loved one) can also elicit positive emotional and brain activation responses [41] and listening to leisure music can support neurocognitive functioning, reduce psychological stress, and encourage sympathetic activation [42]. However, perception of human sounds is ambivalent and context-specific. For instance, in a time of home confinement, prolonged exposure to these sounds may cause excessive sensory input and sense of crowding, which is detrimental to health and well-being due to a lack of privacy [43,44,45]. A Canadian study among people in a home office during the COVID-19 lockdown found that noise coming from occupants in the same suite (i.e., roommates and family) was the biggest issue [46]. Evidence in children suggests that noise inside the home might be one of the mechanisms explaining the negative effect of crowding on well-being [47]. Additionally, of note, individual differences, such as noise sensitivity, can modify sound perception and aggravate subjectively recognizable negative reactions (e.g., dissatisfaction, annoyance, frustration, and anger) [48].

Understanding health effects of these various forms of sound in an unparalleled situation as the COVID-19 lockdown is not straightforward. Insight into how positive soundscape components (e.g., nature sounds) can offset detrimental effects of unwanted sounds (e.g., mechanical sounds) and how ambivalent human sounds fit in can inform acoustic design and enhance human experience when the capacity to avoid or control the acoustic environment is limited. However, we are not aware of earlier epidemiological research that has specifically explored the joined health impact of various forms of sound during the COVID-19 pandemic in residential homes of participants.

**Figure 1:**
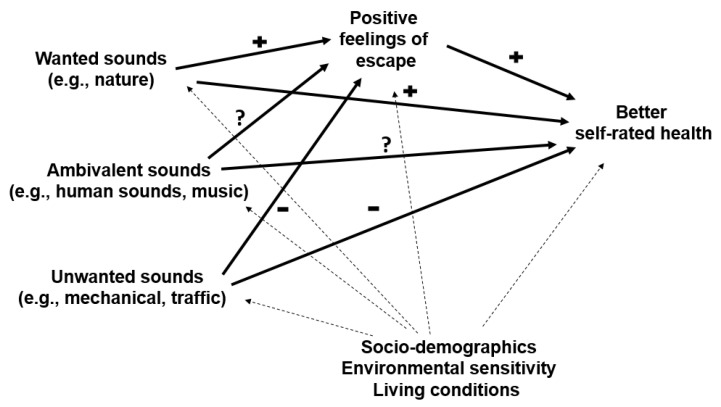
Conceptual framework showing hypothesized pathways between perceived sounds in the home and self-rated health during home confinement. Note: Dashed lines represent hypothesized confounding or modifying effects of contextual factors.

In the present study, we seek to understand how the indoor soundscape related to university students’ self-rated health in Bulgaria around the time that the country was under a state of emergency declaration caused by the COVID-19 pandemic. For this purpose, we use a perceived soundscape approach [49,50] taking up on recent developments in indoor soundscape research [51,52]. First, we explore whether preferences for various forms of sound experienced indoors can be grouped into conceptual categories. Second, we investigate whether exposure to unwanted sounds is associated with worse self-rated health, and exposure to wanted sounds, with better self-rated health. Third, we hypothesize that these effects are carried out by constraining or promoting the feeling of “being away” in the home. This conceptual framework is depicted in Figure 1.

## 2. Materials and Methods

### 2.1. Study Design and Sampling

Between 17 May and 10 June 2020, we conducted a cross-sectional online survey among students from two universities in the city of Plovdiv, Bulgaria. The weeks preceding data collection overlapped both the period of more restrictive measures in Bulgaria, when recreational establishments were closed and access to public outdoor spaces such as parks was banned, and the weeks that followed immediately after, when those measures were gradually relaxed. Students in medicine, dentistry, and biology were approached by their lecturers with an invitation to participate in a survey on living conditions and mental health. Students could also forward the link to their peers. We targeted students in health-related programs because the psychological wear and tear of the student occupation more than likely took a heavy toll on their mental health [53]. To be included, students had to be aged from 18 to 35 years and, to ensure that they were familiar with their neighborhood environment, had to have lived in their current home for at least six months. Further details have been reported elsewhere [10].

The design and conduct of the study followed the general principles outlined in the Declaration of Helsinki. After reading the information about the objectives of the study and instructions on filling-in the questionnaire, all respondents confirmed that they were at least 18 years old and provided informed consent in the survey form, thereby agreeing that their personal information would be processed and stored according to the General Data Protection Regulation in the European Union. The generic design of earlier studies in this series has been approved by the Ethics Committee at the institution of the principal investigator [54].

### 2.2. Sefl-Rated Health

Self-rated health (SRH) was measured with the question “In general, what would you say your health was in the past two weeks?”. Response options included “very poor”, “poor”, “fair”, “good”, and “very good” [55]. Single-item SRH captures various health-relevant factors and states and is considered a good proxy for general health across diverse populations [56,57,58,59].

### 2.3. Perceived Acoustic Environment

Participants were asked how frequently they heard the following types of sounds while at home in the past two weeks: traffic noise (e.g., cars, buses, airplanes, and trains); other mechanical sounds coming from outside (e.g., sirens, construction, and machines); nature sounds coming from outside (e.g., singing birds, flowing water, wind blowing, and rustling leaves); mechanical sounds from within the dwelling (e.g., appliances, pluming, and elevators); music from within the dwelling; and sounds from human beings within the dwelling (e.g., conversation, laughter, children at play, and footsteps). Response options were “0 = never”, “1 = sometimes”, “2 = half of the time”, “3 = most of the time”, and “4 = all the time”.

Next, participants indicated their general preference for these sounds. Response options were “1 = very unpleasant”, “2 = unpleasant”, “3 = neither pleasant nor unpleasant”, “4 = pleasant”, and “5 = very pleasant”. Pleasantness ratings were used to identify conceptual categories of sounds.

In addition, we computed pleasantness-weighted exposure scores as the product of exposure frequency and corresponding pleasantness. To that end, pleasantness ratings were recoded, so that both higher and lower scores indicated greater perceived intensity (positive or negative) of the sound (i.e., “2 = very unpleasant”, “1 = unpleasant”, “0 = neither pleasant nor unpleasant”, “1 = pleasant”, and “2 = very pleasant”). Thus, a higher score reflects a more frequent exposure to a sound that holds greater emotional valence for the individual and vice versa.

### 2.4. Mediator

Restorative quality of the home, and specifically the feeling of “being away” (i.e., opportunity to get distance from daily stressors and routines), was hypothesized to mediate the effect of perceived sounds on SRH. To reduce questionnaire length and response burden, we used a single item [60,61] adapted from the Perceived Restorativeness Scale [62,63]. The questionnaire instructions were adapted to refer to the home environment (i.e., “At home, the time spent gives me a break from my day-to-day routine and I can get away from the things that usually demand my attention.”). Responses were again given on an 11-point scale with two anchors (“0 = not at all” to “10 = completely”).

### 2.5. Confounders

We selected a parsimonious set of variables that could confound or modify the associations between perceived sounds and health based on previous research findings.

Sociodemographic characteristics included age, gender, ethnicity (Bulgarian or not), and income. Income adequacy was measured with a single item: “Having in mind the total monthly income you can make use of, how easy is it for you to meet your expenses without depriving yourself?” Response options ranged from “0 = very difficult” to “5 = very easy”.

House-related characteristics included dwelling type (i.e., apartment, house, or hostel), presence of a quiet room (far from noise sources) and soundproof windows, and crowding in the household (people-to-rooms ratio). We also asked about duration of residence and time spent at home per day.

Noise sensitivity, and by extension general sensitivity to environmental influences [48], may be independently associated with health or modify the effect of noise on health [64,65,66]. Moreover, it may influence perceptions of the acoustic environment, with more sensitive individuals being more likely to be hypervigilant [67,68]. Therefore, we collected data on sensitivity to noise, light, odors, weather conditions, and dust. Participants were asked “Compared to most people, to what extent are you sensitive to each of the following?”. Response options were “0 = not at all sensitive”, “1 = slightly sensitive”, “2 = moderately sensitive”, “3 = very sensitive”, and “4 = extremely sensitive”. We computed the sum of item responses, which could range from 0 to 20, with a higher summary score indicating greater environmental sensitivity. The scale’s internal consistency was not outstanding (McDonald’s ω = 0.65).

Since mental health may modify noise perception and its relationship with health [69], we also measured severities of depressive and anxiety symptoms over the past two weeks. The Patient Health Questionnaire 9-item (PHQ-9) measures the frequency of symptoms of depression [70]. Response options included “0 = not at all”, “1 = several days”, “2 = more than half of the days”, or “3 = nearly every day”. Scores (sum of the item responses) could range from 0 to 27 (McDonald’s ω = 0.68). The Generalized Anxiety Disorder 7-item (GAD-7) scale was used to assess common symptoms of anxiety [71]. Response options were provided on the same scale. Scores (sum of the item responses) could range from 0 to 21. The seven items loaded onto one latent factor and the internal consistency in our sample was high (McDonald’s ω = 0.91). The PHQ-9 and GAD-7 scales were dichotomized, where scores of 10 or above were consistent with moderate depression [72] and generalized anxiety disorder [73], respectively.

We did not have information on the exact home address but did collect the settlement where the respondent currently lived. We classified these settlements as cities (>100,000 residents), towns (10,000 to 100,000), or villages (<10,000). Finally, we retrieved data on which university the respondent studied at.

### 2.6. Analysis Strategy

Missing values (<10% on any given variable) were missing completely at random and imputed using the expectation-maximization algorithm [74]. All variables in multivariate analysis models were included in the imputation. The sound pleasantness ratings were not multivariate normal according to Small’s omnibus test of multivariate normality (VQ3 _(12)_ = 185.90, *p* < 0.001), “being away” had excessive kurtosis, and SRH was left-skewed and treated as ordered categorical. We examined general patterns of associations within the data, using Spearman correlation and Mood’s median test.

As a next step, we conducted an exploratory factor analysis (EFA) to evaluate the dimensionality of the set of sound pleasantness ratings. Procedure to determine the appropriate number of factors to be extracted was parallel analysis [75,76]. The item scores were treated as ordered-categorical variables, so we chose to compute the factor analysis on the basis of polychoric interitem correlations. A factor solution was fitted using robust diagonally weighted least squares (DWLSs) extraction technique. To achieve factor simplicity we used robust promin rotation [77]. Factor loadings ≥ 0.40 were interpreted as salient [78]. Analyses were carried out in FACTOR v. 10.10.02 [79,80].

Then, we tested multivariate associations between mechanical sounds (traffic noise, outdoor, and indoor mechanical sounds), human sounds (human sounds and music), and nature sounds, on the one hand, and “being away” and SRH, on the other. We fitted a robust linear regression model for “being away” and an ordered logistic regression for SRH. We report two types of adjusted models: a main model adjusted for core confounders (age, gender, ethnicity, income, and environmental sensitivity) and a full model additionally adjusted for dwelling type, settlement type, university, presence of a quiet room, soundproof windows, crowding, duration of residence, and time spent at home. As a sensitivity analysis, these models were also fitted by regressing the outcomes on the pleasantness-weighted sound exposure scores instead of the original exposure frequency scores. Tolerance values > 0.2 [81] and variance inflation factor values < 5.0 [82] indicated no multicollinearity.

Stratified analysis and multiplicative interaction terms were computed to investigate possible modification of the associations of mechanical, human, and nature sounds with SRH. Based on earlier related work [83,84,85,86] and literature reviews [87,88], we tested the following a priori modifiers: gender, income, presence of a quiet room, soundproof windows, garden, presence of a terrace/balcony, crowding, environmental sensitivity, depression and anxiety, and time spent at home per day. Criterion for statistical consideration was relaxed to *p* < 0.1 to report relevant effect modification that might otherwise remain undetected [89,90,91].

This was followed up with a structural equation modeling (SEM) to test the theoretically indicated interplay between the variables (Figure 1). We treated SRH as an ordered categorical variable and employed a DWLS estimator [92] with bootstrap-generated (5000 draws) standard errors and confidence intervals for all paths [78,93]. Model specification was guided by theory and bivariate correlations in the dataset. Covariances between the three sound types were specified a priori. Goodness-of-fit was evaluated using indices of acceptable model fit provided in Hu and Bentler [94]: a non-significant χ^2^ (*p* > 0.05); a comparative fit index (CFI) ≥ 0.95; a root mean square error of approximation (RMSEA) ≤ 0.06 with a 90% CI ≤ 0.06; and a standardized root mean square residual (SRMSR) ≤ 0.08. Modification indices were used to identify sources of misfit and, if the suggested modification was supported by theory, the initial model was revised accordingly. Confounding paths with at least marginal statistical significance (*p* < 0.1) were retained in the final solution. An indirect effect (i.e., a product of coefficients for the constituent paths) that significantly exceeded zero was taken as evidence of mediation [95,96]. While we use terminology accepted in mediation modeling to denote the overall (total effect), direct (direct effect), and indirect (indirect effect) relationships in the SEM, the word “effect” should not be taken to indicate claims of causality.

Data were processed Stata v. 14 (StataCorp. 2015. Stata Statistical Software: Release 14. College Station, TX: StataCorp LP.) and the lavaan v. 0.6-7 package [97] for R v. 4.0.3. (R Core Team (2020). R: A language and environment for statistical computing. R Foundation for Statistical Computing, Vienna, Austria.). A *p*-value of < 0.05 was considered statistically significant except as noted above.

## 3. Results

### 3.1. Participant Characteristics

The majority of participants was in their late teens/early twenties, more often female, and Bulgarian. Most participants reported good or very good SRH. Detailed participant characteristics are shown in Table 1.

### 3.2. Exploratory Factor Analysis of Sounds Pleasantness

Several well-recognized criteria for adequacy of the polychoric correlation matrix were used. Bartlett’s test of sphericity was significant (χ^2^_(15)_ = 628.10, *p* < 0.001). The interitem correlation matrix had “fair” sample adequacy (Kaiser–Meyer–Olkin = 0.70; 95% CI: 0.66, 0.76). Parallel analysis indicated that two factors could be extracted to explain the correlations among the six items, and they were labeled according to their content. Pleasantness of traffic noise, outdoor, and indoor mechanical sounds loaded highly on one latent factor, which we labeled “Mechanical sounds”. Pleasantness of human sounds, music, and nature sounds loaded on a second factor, which we labeled “Non-mechanical sounds”. These two factors were moderately correlated (r = 0.37). The initial eigenvalues showed that the first factor explained 43% and the second factor 25% of the variance in the data. Interitem correlations, factor loading matrix, and extraction communalities for the final two-factor solution are presented in Table 2. Most items had high primary loadings and trivial cross-loadings. However, compared with pleasantness of human sounds and music, pleasantness of nature sounds was weakly associated with the factor “Non-mechanical sounds” (<0.40) and had low communality (<0.30). This supported the treatment of nature sounds as semantically different from anthropogenic sounds, as in earlier soundscape research [98,99,100]. Based on these results, we constructed three exposure variables for subsequent analyses: mechanical sounds (traffic noise, outdoor, and indoor mechanical sounds), human sounds (human sounds and music), and nature sounds.

### 3.3. Bivariate Associations

Correlations between the variables are shown in Table 3. SRH was associated with less frequent exposure to mechanical sounds but not with other sounds. Male participants, those with low environmental sensitivity, those who experienced stronger feelings of “being away”, who had a garden, and a quiet room or soundproof windows reported better SRH. Depression and anxiety symptoms were consistent with worse SRH.

“Being away” was higher in older age, in participants having a garden, access to a quiet room, and soundproof windows. “Being away” was inversely related to depression and anxiety symptoms.

Higher environmental sensitivity was related to more frequent perceived exposure to all sound types. Mechanical sounds were negatively associated with “being away”, while nature sounds contributed to “being away”. Participants having a domestic garden reported lower exposure to mechanical and more frequent exposure to nature sounds. On the other hand, crowding related to mechanical and human sounds. Exposure to mechanical sounds was lower in those having a quiet room and those who did not report pronounced depressive and anxiety symptoms. From Table 4, participants living in a village were less frequently exposed to mechanical and human sounds. Apartment dwellers reported lower exposure to nature sounds, and house dwellers, to mechanical sounds.

### 3.4. Multivariate Associations between Perceived Sounds Exposure and Self-Rated Health

In the main model, nature sounds were positively associated with “being away” (Table 5). “Being away” was also associated with older age, higher income adequacy, and lower environmental sensitivity. These findings persisted in the full model.

Only mechanical sounds were associated with worse SRH. The estimates associated with human and nature sounds were non-significant (Table 5). In addition, lower environmental sensitivity and higher income related to better SRH. In the full model, controlling for additional covariates did not materially change the effect sizes for perceived sounds sound, but having soundproof windows was independently associated with better SRH.

These patterns of association were also observed in the sensitivity analysis, in which pleasantness-weighted sound exposure scores were used. Respective effect estimates in the main model were OR = 0.91 (95% CI: 0.88, 0.95) for mechanical sounds, OR = 1.01 (95% CI: 0.95, 1.08) for human sounds, and OR = 1.03 (95% CI: 0.96, 1.12) for nature sounds.

### 3.5. Effect Modification of the Association between Perceived Sounds Exposure and Self-Rated Health

Table 6 shows the results of stratified analysis. Few of the tested interactions were statistically significant at the *p* < 0.10 level. Mechanical sounds were more strongly associated with worse SRH in less crowded household and when participants experienced depression. Human sounds were associated with better SRH in participants without soundproof windows installed, while the effect size itself was statistically significant only in men. The effect of nature sounds went in a positive direction when participants did not have a domestic garden and in crowded households.

### 3.6. Structural Equation Model of the Effect of Perceived Sounds Exposure, as Mediated by “Being Away”, on Self-Rated Health

Goodness-of-fit levels for the initial model were not outstanding: χ^2^_(22)_ = 40.92, *p* = 0.008; CFI = 0.90; RMSEA = 0.05 (90% CI: 0.03, 0.08); and SRMR = 0.05. Therefore, the model was modified by removing paths associated with a *p*-value larger than 0.10. Ethnicity was dropped from the model.

**Figure 2:**
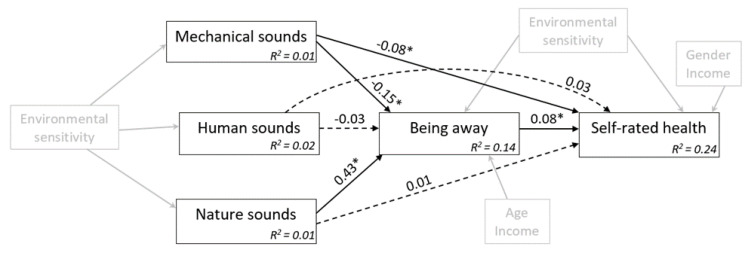
Structural equation model showing the estimated paths linking perceived sounds to feelings of “being away” and self-rated health. Note: Unstandardized regression weights with their significance level are given for each path. R^2^ shows proportion of variance explained in endogenous variables. Coefficients marked with an asterisk (*) are statistically significant at *p* < 0.05. Control variables are shown in grey. Covariances, associations between control variables, and errors terms are not displayed to enhance readability.

Goodness-of-fit statistics associated with the modified model indicated good consistency with the data: χ^2^_(19)_ = 18.86, *p* = 0.466; CFI = 1.00; RMSEA = 0.00 (90% CI: 0.00, 0.05); and SRMR = 0.09. The model explained 22% of the variance in SRH and 14% in “being away”. As can be seen in Table 7 and Figure 2, only mechanical sounds were associated with a significant total effect on SRH. On the other hand, both mechanical and nature sounds had indirect effects on SRH through “being away”. These indirect pathways operated in opposite directions—mechanical sounds related to weaker feelings of “being away”, and then to worse SRH; conversely, nature sounds were associated with stronger feelings of “being away”, and in turn with better SRH.

## 4. Discussion

### 4.1. General Findings

This study explored associations between different forms of sound experienced indoors and self-rated health during the COVID-19 quarantine in Bulgaria when students spent home almost all of their time. In the multivariate regression analyses, greater exposure to mechanical sounds was consistently associated with worse SRH, whereas no significant associations were found for human and nature sounds. However, SEM revealed interesting mechanistic insights. While a total effect was only observed for mechanical sounds, supporting regression-based findings, both mechanical and nature sounds were found to indirectly relate to SRH through “being away”, that is, the perceived restorative capacity of the home environment. Exposure to mechanical sounds related to lower restorative quality of the home, and then to poorer SRH, whereas nature sounds correlated with higher restorative quality, and in turn with better SRH.

Exploration of the dimensionality of perceived pleasantness via EFA supported grouping individual sound exposures into three distinct factors—mechanical, human, and nature sounds. This factorial structure is consistent with categorization of sounds in soundscape studies [98,99,100] and also reflects differential affective response to sounds and underlying neural networks [101,102]. Earlier research has identified distinct neural activation patterns corresponding to perception of these conceptual categories of sounds, with human sounds yielding activation preferentially in the bilateral posterior superior temporal sulci, animal sounds—in the bilateral posterior insulae, and mechanical sounds—in the anterior superior temporal gyri and parahippocampal cortices [101]. Albeit distinct in terms of perceived pleasantness, human and nature sounds stood closer together relative to mechanical sounds, in line with the broader cognitive differentiation between “living” and “non-living” sounds [101,103].

Our findings about mechanical sounds align with a large body of evidence linking traffic noise to a variety of ill-health outcomes [104]. Fewer studies used health related quality of life [105,106,107] or poor self-rated health, as operationalized here [108,109]. Even less is known about how perceived soundscape affects self-rated health in residential homes [87,110], as most studies of this type were conducted in public places like parks or shopping areas, where people spend only a small amount of time compared with the time spent home. While there is no doubt that unwanted sounds, such as traffic noise, can directly affect health and quality of life, it often escapes the spotlight that noise can in addition constrain psychophysiological stress recovery in a setting [111]. This indirect effect can lead to worse adaptation to other concurrent environmental stressors [6], which in turn may be further compounded with the already constrained restoration of student participants attending online lessons at home (cf. [7,8,9,10]).

Nature sounds did not directly result in better SRH, but the presence of a total effect is not a prerequisite for a meaningful indirect effect to exist [96]. Nature sounds seemed to work through restorative quality, which is consistent with a previously reported contribution of bird songs [37] and other nature sounds [112,113] to stress recovery and perceived attention. Stress reduction theory holds that multimodal sensory input from nature can evoke positive emotions and block psychophysiological stress as a result of evolutionary embedded preference for natural attributes, such as sounds and visual configurations [114,115]. In addition, according to Attention restoration theory, natural environments readily enable recovery of fatigued attentional resources needed to cope with environmental stressors [116,117]. Auditory natural stimuli are a key component of nature experience and may even drive stress recovery beyond visual exposure [112]. Hence, nature sounds penetrating the dwelling may provide rich information content and act as sensory cues retrieving relaxation conditioned on the positive affect and tranquility experienced in natural settings (cf. [118,119]). An alternative explanation of our findings could be wanted sounds enhancing indoor soundscape pleasantness and eventfulness through informational masking of unwanted sounds [120,121,122]. However, the masking effect is greater when the competing stimuli are the same frequency, and most nature sounds tend to be too high-pitched compared with low frequency components of mechanical sounds [123,124].

In the stratified analysis, there was some indication of potentially beneficial associations, but no clear pattern was observed for human sounds. We are mindful that a lack of specificity in our items could have introduced bias, especially with regard to ambivalent human sounds, which may activate processes of either stress or restoration, depending on their affective content [102]. For example, a baby’s cry will have lower valence and instigate greater arousal than a baby’s laugh [102]. It could be that, since we did not differentiate between sound sources, positive (e.g., voices of loved ones) and negative (e.g., neighbor sounds) semantic components were both captured by the items and cancelled each other out. On the one hand, noises coming from neighbors can be especially annoying while students are trapped in their dwelling and need peace and quiet to focus on their studies. Conversely, presence of human sounds can reinforce feelings of social embeddedness, which is an essential resource in times of isolation from one’s usual social network (cf. [125]).

Overall, our findings support the importance of indoor soundscape for shaping perceived health through the extent to which the home environment is conducive to psychological restoration. A restorative environment, such as one supplying nature sounds and relatively free of mechanical sounds, could contribute to personal coping resources needed to offset stress [6,126].

### 4.2. Secondary Findings

Among the other factors investigated here, environmental sensitivity emerged as highly predictive of worse SRH. Although not yet fully understood, noise sensitivity is a psychometric construct with underlying neurobiological and genetic components [127], which represents individual’s heightened reactivity to auditory stimuli in general [128]. It partly overlaps with other types of environmental sensitivity [63,128] and has been found to independently contribute to psychological and physical ill-health [129,130], and modify the effects of noise on health [131,132,133]. However, overall results are inconsistent [134], which may in part explain why we observed no effect modification by environmental sensitivity. Income adequacy was also predictive of “being away” and SRH, which is not surprising since perceived income affects the probability of reporting a better health more than objective income [135].

By and large, evidence of effect modification in our study was sparse. The effect of mechanical sounds was robust across various individual and contextual factors, with statistical evidence of effect modification by crowding and depression. That is, low crowding and presence of depressive symptoms seemed to compound the negative effect. While crowding is usually considered detrimental to health and well-being [136], having more people in the household in a time of loss of other immediate social interaction may be protective or alter processing of mechanical sounds by providing distraction. As for depressed individuals, they may have a more pronounced annoyance reaction to noise [69]. Concerning human sounds, we only saw effect modification by having soundproof windows, but we can offer no satisfactory explanation. This finding could be the result of unaccounted for confounding or merely a statistical artifact. We suspect that a lack of specificity in the soundscape items and referring to sounds inside the dwelling in general could have obscured effect modification by access to a quiet room. In participants having a domestic garden, nature sounds did not appear protective, arguably because they may not have felt mobility restrictions as much as those without gardens and could spend unrestricted amounts of time outdoors, which would itself drive the positive health effect (as suggested by bivariate correlations). Conversely, for those who lacked access to an actual natural setting, nature sounds probably served as a surrogate. The finding that in crowded households nature sounds were associated with better SRH is difficult to explain and may again have to do with the intertwining of contextual factors and soundscape perception. However, our data were not suitable for exploring higher order interactions.

### 4.3. Strengths and Limitations

To our knowledge, the current study was the first to explore health effects of various forms of perceived sounds while people were forced to spend unprecedented amount of time in their homes. We also tested for the first time indirect pathways linking soundscape to health in residential environments through feelings of “being away”, one important component of a person’s environmental restorative quality at home. We collected data on both exposure frequency and pleasantness. This allowed us to explore the underpinning dimensionality of sound preference, which guided the differentiation between wanted and unwanted soundscape components. Finally, we accounted for multiple contextual (i.e., income and housing characteristic) and individual factors (i.e., environmental sensitivity and mental health), which could have confounded or modified the effects of soundscape on self-rated health.

Several limitations should also be acknowledged. Our sample size was modest and, therefore, especially our ordinal regression models could have lacked sufficient power to detect small total effects of human and nature sounds. In this vein, ratings of perceived soundscape and “being away” were narrowly distributed and little variation in these variables could have attenuated or even concealed potentially meaningful associations despite our use of robust estimation techniques. We used a convenience sample of students in health-related programs, which was restrictive as to participants’ economic and non-professional activity, precluding generalization of findings to experiences of all youth during the COVID-19 epidemic. Moreover, an actual response rate could not be calculated given that students were also allowed to forward the survey link to their peers.

Next, a cross-sectional design does not allow making causal claims about the associations observed. It is possible that participants’ health status determined their environmental awareness and salience of sounds. We attempted to control for this by introducing environmental sensitivity and mental health into the models. Reassuringly, the associations of interest were robust to confounding/effect modification by those factors. Furthermore, cross-sectional analyses of mediation may yield findings even in the absence of an indirect effect [137,138]. Still, our predictor, mediator, and outcome variables, even if measured at one point in time, satisfied the conceptual timing criterion (i.e., conceptual time-ordering) required for mediation [139].

Further, we only collected data on perceived health and soundscape. SRH has been found to agree reasonably well with physician-diagnosed health status and be a good predictor of adverse health outcomes in earlier studies [56,57,58,59]. However, we could not ascertain the predictive validity of SRH in our sample and we acknowledge that biomedical data used to establish the validity of SRH may vary over time, social, and age strata. In addition, we chose to focus on perceptual soundscape measures, owing to a lack of data on participant’s residential address and the large uncertainty in expected sound exposures at the receiver’s home. Out of privacy concerns, we did not collect information on participant’s residential address, which thwarted linkage to existing noise maps. Even if such data were available, we could not rely on the official noise maps of Plovdiv, because of the profound and spatially variable changes observed in traffic flow and structure during the lockdown [23,140] and the fact that only half of the participants resided in Plovdiv in the period of interest. Moreover, typical average noise level measurements would not only underestimate participants’ actual exposure to all sources at all sites both indoors and outdoors, but also carrying out measurements at participants’ homes was not feasible during the state of emergency. We acknowledge that using psychoacoustic noise indicators would have allowed for better discrimination of sound pleasantness and other perceptual dimensions [30,52] and raised our confidence in the associations observed.

Finally, while we collected data on additional housing and lifestyle factors (e.g., exposure to green spaces, presence of pets in the household, and physical activity) [10], we deliberately used only a subset of these variables for the current study. Given that all these data were self-reported, there is a high potential for common-source bias and/or reverse causation, which would thwart interpretation of a more complex structural model. We also deliberately focused on a more narrowly defined model since the nature and quality of our data did not allow for strong inferences about more complex structures where “being away” would assume the lead role as a central link mediating competitive indirect pathways leading to SRH.

## 5. Conclusions

Sounds perceived by university students in their home during their COVID-19 quarantine were conceptually grouped into three distinct factors: mechanical, human, and nature sounds. Greater exposure to mechanical sounds was consistently associated with both lower restorative quality of the home and worse self-rated health. Apart from these observed direct effects, exposure to mechanical sounds related to lower perceived restorative quality of the home, and then to worse self-rated health, whereas nature sounds were associated with stronger feelings of escape, and in turn with better self-rated health. No association was found with human sounds. These findings suggest a role of positive indoor soundscape and restorative quality for promoting self-rated health in times of social distancing.

## Figures and Tables

**Table 1:** Participant characteristics (*N* = 323).

Characteristic	Statistic	Range
**Socio-demographics**		
Age (median years, IQR)	21.00 (3.00)	18.00–35.00
Male (*N*, %)	100 (31.0)	
Bulgarian (*N*, %)	281 (87.0)	
Income adequacy (mean, SD)	3.24 (1.11)	0.00–5.00
Self-rated health (*N*, %)		
Very poor	3 (0.9)	
Poor	16 (5.0)	
Fair	83 (25.7)	
Good	137 (42.4)	
Very good	84 (26.0)	
**Perceived exposure (median, IQR)**		
Mechanical sounds	4.00 (4.00)	0.00–12.00
Human sounds	3.00 (3.00)	0.00–8.00
Nature sounds	3.00 (3.00)	0.00–4.00
**Mediator**		
Being away (median, IQR)	5.00 (5.00)	0.00–10.00
**Confounders/modifiers**		
Depression (*N*, %)	112 (34.7)	
Anxiety (*N*, %)	70 (21.7)	
Environmental sensitivity (mean, SD)	9.87 (3.79)	0.00–20.00
Dwelling type (*N*, %)		
Apartment	191 (59.1)	
House	120 (37.2)	
Hostel	12 (3.7)	
Duration of residence (median years, IQR)	14.00 (16.00)	0.50–32.00
Time at home (median hrs/day, IQR)	20.00 (4.50)	7.50–24.00
Crowding (median, IQR)	1.00 (0.58)	0.17–5.00
Settlement type (*N*, %)		
City	160 (49.5)	
Town	136 (42.1)	
Village	27 (8.4)	
University (*N*, %)		
Medical University of Plovdiv	241 (74.6)	
Plovdiv University	82 (25.4)	

Abbreviations: IQR—interquartile range, SD—standard deviation.

**Table 2:** Statistics for the sounds pleasantness items in the two-factor exploratory factor analysis rotated solution.

Sounds Pleasantness	Factor Loadings (95% CI) ^1^		EC	Mean (SD)	Polychoric Correlations					
	F1: Mechanical	F2: Non-Mechanical			1.	2.	3.	4.	5.	6.
1. Traffic	0.76 (0.64, 0.85)		0.55	2.11 (0.90)	1.00					
2. Indoor mechanical	0.77 (0.66, 0.84)		0.66	2.08 (0.81)	0.57	1.00				
3. Outdoor mechanical	0.93 (0.85, 0.98)		0.81	1.78 (0.77)	0.66	0.70	1.00			
4. Music		0.82 (0.69, 0.91)	0.63	3.59 (1.08)	0.12	0.23	0.13	1.00		
5. Human		0.78 (0.66, 0.87)	0.61	3.15 (1.08)	0.16	0.29	0.20	0.61	1.00	
6. Nature		0.34 (0.15, 0.49)	0.12	4.41 (0.78)	0.08	0.14	0.10	0.27	0.28	1.00

Abbreviations: EC—Extraction communality, SD—Standard deviation. ^1^ Loadings < 0.30 are not displayed.

**Table 3:** Spearman correlations between the main variables in the study.

Variable	(1)	(2)	(3)	(4)	(5)	(6)	(7)	(8)	(9)	(10)	(11)	(12)	(13)	(14)	(15)	(16)	(17)	(18)	(19)
(1) Self-rated health	1.00																		
(2) Mechanical sounds	−0.26 ^1^	1.00																	
(3) Human sounds	−0.02	0.24 ^1^	1.00																
(4) Nature sounds	0.04	−0.04	0.21 ^1^	1.00															
(5) Gender (female vs. male)	−0.14 ^1^	0.01	0.07	0.01	1.00														
(6) Age	0.07	0.07	−0.03	−0.10	−0.04	1.00													
(7) Ethnicity (other vs. Bulgarian)	−0.06	0.06	0.07	0.11 ^1^	0.14 ^1^	−0.10	1.00												
(8) Income adequacy	0.20 ^1^	−0.07	0.10	0.05	−0.04	0.12 ^1^	0.05	1.00											
(9) Environmental sensitivity	−0.23 ^1^	0.19 ^1^	0.10	0.12 ^1^	0.15 ^1^	−0.06	0.05	−0.09	1.00										
(10) Being away	0.32 ^1^	−0.18 ^1^	−0.06	0.15 ^1^	0.04	0.17 ^1^	−0.02	0.19	−0.18 ^1^	1.00									
(11) Crowding	0.03	0.15 ^1^	0.24 ^1^	0.01	0.06	−0.04	0.10	−0.09	0.01	−0.08	1.00								
(12) Garden	0.12 ^1^	−0.37 ^1^	−0.07	0.21 ^1^	0.00	−0.13 ^1^	0.04	0.02	−0.05	0.14 ^1^	−0.22 ^1^	1.00							
(13) Terrace/balcony	0.10	0.01	0.01	−0.04	−0.12 ^1^	0.08	−0.08	0.03	−0.09	0.07	−0.07	−0.25 ^1^	1.00						
(14) Quiet room	0.18 ^1^	−0.34 ^1^	−0.05	0.09	−0.03	0.04	−0.05	0.14 ^1^	−0.12 ^1^	0.20 ^1^	−0.18 ^1^	0.23 ^1^	0.06	1.00					
(15) Soundproof windows	0.21 ^1^	−0.10	−0.00	0.09	−0.05	0.16 ^1^	−0.04	0.22 ^1^	−0.00	0.15 ^1^	−0.12	0.04	0.03	0.34 ^1^	1.00				
(16) Depression	−0.48 ^1^	0.17 ^1^	0.03	0.02	0.16 ^1^	−0.12 ^1^	0.12 ^1^	−0.09	0.28 ^1^	−0.24 ^1^	0.01	−0.03	−0.15 ^1^	−0.14 ^1^	−0.15 ^1^	1.00			
(17) Anxiety	−0.44 ^1^	0.18 ^1^	0.09	−0.02	0.06	−0.11 ^1^	−0.02	−0.05	0.18 ^1^	−0.25 ^1^	−0.05	−0.04	−0.07	−0.15 ^1^	−0.14 ^1^	0.56 ^1^	1.00		
(18) Duration of residence	0.09	−0.05	0.06	0.05	−0.10	0.08	−0.02	0.06	−0.09	0.00	0.02	0.16 ^1^	0.03	0.02	0.01	−0.12 ^1^	−0.01	1.00	
(19) Time at home/day	−0.11	0.09	−0.08	0.10	0.02	0.03	0.12 ^1^	−0.03	0.10	0.01	0.02	−0.02	−0.08	−0.12 ^1^	−0.13 ^1^	0.23 ^1^	0.15 ^1^	−0.05	1.00

^1^ Correlation is statistically significant at *p* < 0.05 level (two-tailed).

**Table 4:** Associations between multicategorical participant characteristics and self-rated health, being away, and perceived sounds.

Characteristics	Being Away	Self-Rated Health	Mechanical Sounds	Human Sounds	Nature Sounds
Dwelling type					
Apartment	5.00 (5.00)	3.00 (1.00)	4.00 (4.00) ^1^	3.00 (3.00)	2.00 (2.00) ^1^
House	6.00 (6.50)	3.00 (2.00)	3.00 (3.00) ^1^	3.00 (2.50)	3.00 (2.00) ^1^
Hostel	5.00 (5.50)	3.00 (1.50)	4.00 (3.50) ^1^	3.00 (2.50)	3.00 (2.00) ^1^
Settlement type					
City	5.00 (5.00)	3.00 (2.00)	4.00 (4.00) ^1^	3.00 (2.00) ^1^	2.00 (2.00)
Town	5.00 (5.50)	3.00 (1.00)	4.00 (4.50) ^1^	3.00 (3.00) ^1^	3.00 (2.00)
Village	6.00 (7.00)	3.00 (2.00)	2.00 (3.00) ^1^	2.00 (2.00) ^1^	3.00 (2.00)

Coefficients are medians with their interquartile range. ^1^ Difference between subgroups is statistically significant, *p* < 0.05 level (two-tailed). *p*-values are from the median test.

**Table 5:** Associations between perceived sounds and “being away” and self-rated health.

Predictors	Being Away		Self-Rated Health	
	Main Model (R^2^ = 0.13)	Full Model (R^2^ = 0.17)	Main Model (R^2^ = 0.06)	Full Model (R^2^ = 0.07)
	β (95% CI)	β (95% CI)	OR (95% CI)	OR (95% CI)
Mechanical sounds	−0.12 (−0.25, 0.01)	−0.06 (−0.20, 0.08)	0.86 (0.79, 0.93) ^1^	0.86 (0.79, 0.94) ^1^
Human sounds	−0.09 (−0.28, 0.10)	−0.07 (−0.27, 0.13)	1.05 (0.94, 1.19)	1.04 (0.91, 1.17)
Nature sounds	0.47 (0.20, 0.74) ^1^	0.41 (0.14, 0.69) ^1^	1.06 (0.89, 1.25)	1.06 (0.88, 1.26)
Gender (female vs. male)	0.57 (−0.18, 1.32)	0.40 (−0.38, 1.18)	0.64 (0.40, 1.01)	0.66 (0.41, 1.06)
Age	0.16 (0.04, 0.28) ^1^	0.16 (0.04, 0.28) ^1^	1.02 (0.95, 1.09)	1.00 (0.93, 1.07)
Ethnicity (other vs. Bulgarian)	−0.26 (−1.33, 0.81)	−0.28 (−1.43, 0.88)	0.80 (0.43, 1.46)	0.92 (0.49, 1.75)
Income adequacy	0.50 (0.16, 0.84) ^1^	0.49 (0.15, 0.83) ^1^	1.29 (1.07, 1.57) ^1^	1.22 (1.00, 1.49)
Environmental sensitivity	−0.14 (−0.23, −0.06) ^1^	−0.15 (−0.24, −0.06) ^1^	0.91 (0.86, 0.97) ^1^	0.92 (0.87, 0.97) ^1^
Settlement type				
Town vs. city		0.28 (−0.56, 1.14)		0.72 (0.43, 1.19)
Village vs. city		0.55 (−0.76, 1.86)		0.66 (0.27, 1.62)
University (PU vs. MUP)		0.60 (−0.33, 1.52)		1.04 (0.61, 1.76)
Dwelling type				
House vs. apartment		0.18 (−0.68, 1.05)		1.03 (0.61, 1.74)
Hostel vs. apartment		−1.06 (−3.32, 1.20)		0.71 (0.23, 2.20)
Duration of residence		−0.03 (−0.07, 0.02)		1.02 (0.99, 1.04)
Time at home/day		0.08 (−0.05, 0.21)		0.98 (0.91, 1.05)
Crowding		−0.19 (−0.89, 0.51)		1.18 (0.75, 1.84)
Quiet room		0.72 (−0.10, 1.53)		1.15 (0.70, 1.89)
Soundproof windows		0.38 (−0.36, 1.11)		1.82 (1.15, 2.88) ^1^

Abbreviations: MUP—Medical University of Plovdiv, PU—Plovdiv University. Coefficients are unstandardized linear regression coefficients (β) and ordered odds ratios (ORs) with their confidence interval (95% CI). ^1^ Point estimate is statistically significant at the *p* < 0.05 level (two-tailed).

**Table 6:** Effect modification of the associations between perceived sounds and self-rated health.

Effect Modifier	*N*	Mechanical Sounds	*p*-int.	Human Sounds	*p*-int.	Nature Sounds	*p*-int.
Gender			0.136		0.154		0.233
Male	100	0.78 (0.67, 0.90)		1.31 (1.02, 1.68)		1.22 (0.88, 1.68)	
Female	223	0.88 (0.81, 0.97)		1.00 (0.87, 1.15)		1.01 (0.82, 1.24)	
Income adequacy			0.674		0.912		0.473
Low	193	0.85 (0.78, 0.95)		1.05 (0.90, 1.22)		1.02 (0.82, 1.28)	
High	130	0.84 (0.73, 0.97)		1.06 (0.87, 1.30)		1.16 (0.87, 1.54)	
Quiet room			0.653		0.882		0.923
No	210	0.87 (0.79, 0.95)		1.04 (0.89, 1.21)		1.05 (0.85, 1.30)	
Yes	113	0.90 (0.76, 1.07)		1.04 (0.84, 1.29)		1.09 (0.80, 1.50)	
Soundproof windows			0.622		0.090 ^1^		0.254
No	183	0.84 (0.76, 0.93)		1.16 (0.98, 1.37)		1.09 (0.87, 1.37)	
Yes	140	0.90 (0.80, 1.02)		0.95 (0.79, 1.13)		0.98 (0.75, 1.28)	
Garden			0.212		0.193		0.028 ^1^
No	171	0.89 (0.80, 0.98)		1.07 (0.92, 1.25)		1.17 (0.93, 1.48)	
Yes	152	0.81 (0.70, 0.93)		1.02 (0.85, 1.24)		0.84 (0.64, 1.11)	
Terrace/balcony			0.201		0.593		0.680
No	46	0.60 (0.43, 0.84)		1.21 (0.84, 1.74)		0.90 (0.51, 1.58)	
Yes	277	0.88 (0.81, 0.95)		1.02 (0.90, 1.16)		1.09 (0.90, 1.31)	
Crowding			0.094 ^1^		0.736		0.012 ^1^
Low	204	0.79 (0.71, 0.88)		1.10 (0.94, 1.28)		0.87 (0.70, 1.09)	
High	119	0.92 (0.82, 1.03)		0.91 (0.74, 1.11)		1.34 (1.00, 1.80)	
Environmental sensitivity			0.567		0.618		0.108
Low	148	0.87 (0.77, 0.98)		1.04 (0.86, 1.24)		0.91 (0.70, 1.17)	
High	175	0.85 (0.77, 0.94)		1.05 (0.89, 1.23)		1.19 (0.94, 1.51)	
Depression			0.007 ^1^		0.293		0.602
No	211	0.95 (0.86, 1.05)		0.95 (0.82, 1.11)		1.07 (0.86, 1.34)	
Yes	112	0.77 (0.67, 0.87)		1.14 (0.93, 1.39)		1.08 (0.81, 1.45)	
Anxiety			0.359		0.700		0.366
No	253	0.90 (0.82, 0.98)		1.06 (0.92, 1.21)		1.00 (0.82, 1.22)	
Yes	70	0.83 (0.71, 0.97)		1.13 (0.87, 1.48)		1.24 (0.83, 1.87)	
Time at home			0.261		0.288		0.770
<20 h	185	0.92 (0.83, 1.02)		0.95 (0.80, 1.12)		1.14 (0.91, 1.43)	
> 20 h	138	0.79 (0.70, 0.89)		1.17 (0.97, 1.41)		1.00 (0.76, 1.32)	

Abbreviations: *p*-int.—significance of interaction term (perceived sounds × effect modifier). Coefficients are ordered odds ratios with their confidence interval (95% CI). All models are adjusted for gender, age, ethnicity, income adequacy, and environmental sensitivity (when the respective covariate is not tested as a modifier). ^1^ Interaction term is statistically significant at *p* < 0.1 level (two-tailed).

**Table 7:** Associations between perceived sounds, “being away” and self-rated health in the structural equation model.

	Being Away		Self-Rated Health	
	β (95% CI)	*p*-Value	β (95% CI)	*p*-Value
**Direct effects**				
Mechanical sounds	−0.15 (−0.28, −0.02)	0.025	−0.08 (−0.12, −0.04)	<0.001
Human sounds	−0.03 (−0.22, 0.16)	0.750	0.03 (−0.03, 0.10)	0.344
Nature sounds	0.43 (0.16, 0.71)	0.002	0.01 (−0.09, 0.11)	0.881
**Indirect effects**				
Mechanical sounds	-	-	−0.01 (−0.03, −0.001)	0.057
Human sounds	-	-	−0.002 (−0.02, 0.01)	0.759
Nature sounds	-	-	0.03 (0.01, 0.06)	0.014
**Total effects**				
Mechanical sounds	−0.15 (−0.28, −0.02)	0.025	−0.09 (−0.13, −0.05)	<0.001
Human sounds	−0.03 (−0.22, 0.16)	0.750	0.03 (−0.04, 0.10)	0.400
Nature sounds	0.43 (0.16, 0.71)	0.002	0.04 (−0.06, 0.14)	0.409

Coefficients are unstandardized regression coefficients (β) with their 95% confidence intervals (CI) and *p*-value.

## Data Availability

The data presented in this study are available on reasonable request from the corresponding author. The data are not publicly available as per participants’ informed consent conditions.
